# Synthesis and antibacterial and antifungal activities of *N*-(tetra-*O*-acetyl-β-d-glucopyranosyl)thiosemicarbazones of substituted 4-formylsydnones

**DOI:** 10.1186/s13065-015-0138-8

**Published:** 2015-10-19

**Authors:** Nguyen Dinh Thanh, Hoang Thanh Duc, Vu Thi Duyen, Phan Manh Tuong, Nguyen Van Quoc

**Affiliations:** Faculty of Chemistry, VNU University of Science, 19 Le Thanh Tong, Hoan Kiem, Ha Noi, Vietnam; Faculty of Chemistry, Hanoi University of Industry, Minh Khai, Tu Liem, Ha Noi, Vietnam; Faculty of Chemistry, Vinh University, 182 Le Duan, Vinh, Nghe An Vietnam

**Keywords:** Antibacterial, Antifungal, d-Glucose, Microwave-assisted synthesis, Sydnones, Thiosemicarbazones

## Abstract

**Background:**

Sydnone is a heterocycle that exhibits remarkable pharmacological activities, including antimicrobial, anti-inflammatory, analgesic, antipyretic and antioxidant activities. Thiosemicarbazones are of compounds that contain the –NHCSNHN=C< linkage group and are considerable interest because they exhibit important chemical properties and potentially beneficial biological activities. Similarly, thiosemicarbazones having carbohydrate moieties also exhibit various significant biological activities.

**Results:**

The compounds of 3-formyl-4-phenylsydnones were obtained by Vilsmeyer-Haack’s formylation reaction and were transformed into thiosemicarbazones by condensation reaction with *N*-(2,3,4,6-tetra-*O*-acetyl-β-d-glucopyranosyl)thiosemicarbazide. Reaction were performed in the presence glacial acetic acid as catalyst using microwave-assisted heating method. Reaction yields were 43‒85 %. The antimicrobial activities of these thiosemicarbazones were screened in vitro by using agar well diffusion and MIC methods. Among these thiosemicarbazones, compounds **4k**, **4l**, **4m** and **4n** were more active against all tested bacterial strains, especially against *S*. *epidermidis*, *B. subtilis* and *E. coli*. The MIC values in these cases are 0.156, 0.156 and 0.313 μg/mL, respectively. All compounds showed weak to moderate antifungal activity against *C. albicans* and *A. niger* than nystatin (MIC = 0.156‒0.625 μg/mL vs. MIC = 0.078 μg/mL of nystatin), and thiosemicarbazones **4l**, **4m** and **4n** exhibited significant activity with MIC = 0.156 μg/mL. These compounds also had good antifungal activity against *F. oxysporum* similarly to nystatin (MIC = 0.156 μg/mL). Among the tested compounds having halogen group **4k**, **4l**, **4m** and **4n** showed highest activity against three strains of fungal organisms.

**Conclusions:**

In summary, we have developed a clean and efficient methodology for the synthesis of novel thiosemicarbazone derivatives bearing sydnone ring and d-glucose moiety; the heterocyclic and monosaccharide system being connected via ‒NH‒C(=S)NH‒N=C< linker using molecular modification approach. The methodology could be further extended and used for the synthesis of other thiosemicarbazones of biological importance. 4-Formyl-3-arylsydnone *N*-(2,3,4,6-tetra-*O*-acetyl-β-d-glucopyranosyl)thiosemicarbazones have been synthesized under microwave-assisted heating conditions. Almost all obtained compounds showed remarkable activities against the tested microorganisms. Among the tested compounds having halogen group **4k**, **4l**, **4m** and **4n** showed highest activity against all tested strains of bacterial and fungal organisms.

**Graphical abstract:**
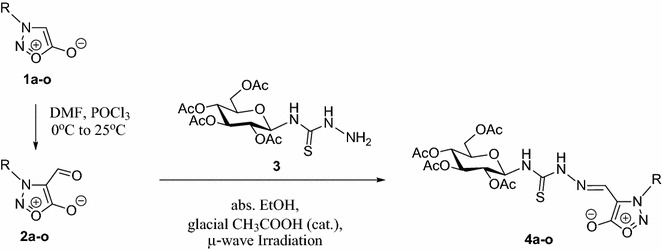
Synthesis and antibacterial and antifungal activities of* N*-(tetra-*O*-acetyl-β-D-glucopyranosyl)thiosemicarbazones of substituted 4-formylsydnones

## Background

Sydnone is a mesoionic aromatic system, which could be described with some polar resonance structures [1]. Several compounds containing a sydnone ring exhibit remarkable pharmacological activities, including antimicrobial, anti-inflammatory, analgesic, antipyretic and antioxidant activities [2–5].

Thiosemicarbazones are compounds that contain the –NHCSNHN=C< linkage group. This class of compounds is of considerable interest because thiosemicarbazones exhibit the important chemical properties and potentially beneficial biological activities [6–9]. Some thiosemicarbazones of 3-aryl-4-formylsydnones were synthesized in good yields by the reactions of 3-aryl-4-formylsydnones with 4′-phenylthiosemicarbazide and thiosemicarbazide, respectively [3, 4]. On the other hand, some monosaccharide thiosemicarbazides are of interested because these derivatives could be used as versatile intermediates for synthesis of various derivatives (especially heterocycles [10]) as well as be used for making complex formations of metallic ions [11, 12].

Thiosemicarbazones having carbohydrate moieties also exhibit various significant biological activities. In recent times, a number of thiosemicarbazones derivatives containing monosaccharide moiety have not yet been synthesized more. In general, thiosemicarbazones derivatives containing monosaccharide moiety have showed remarkable anti-microorganism and antioxidant activity both in vivo and in vitro [13–15]. Some articles have been reported about the synthesis of substituted aromatic aldehyde/ketone *N*-(per-*O*-acetylated glycopyranosyl)thiosemicarbazones in the past [10, 13–15]. These compounds have been synthesized by reaction of *N*-(per-*O*-acetylglycosyl)thiosemicarbazides with the corresponding carbonyl compounds [10, 13, 16–24], but the thiosemicarbazones containing both monosaccharide and sydnone moieties have not been reported yet. Continuing the previous studies on the synthesis and the reactivity of *N*-(per-*O*-acetyl-d-glycopyranosyl)thiosemicarbazides [15, 24], we report in the present paper a study on the synthesis, spectral characterization, antibacterial and antifungal activity of a series of *N*-(tetra-*O*-acetyl-β-d-glucopyranosyl)thiosemicarbazones having sydnone moiety by using microwave-assisted heating method [25].

## Results and discussion

### Chemistry

**Scheme 1:**
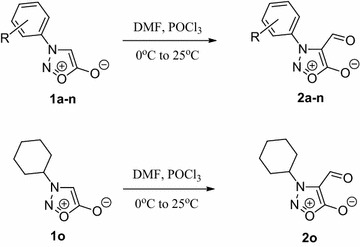
Synthetic pathway for 3-aryl-4-formylsydnones **2a-n** and 3-cyclohexyl-4-formylsydnone **2o**

Required substituted 4-arylsydnones **1a**–**o** [26, 27] and 3-aryl-4-formylsydnone **2a**–**o** [28, 29] were prepared with some modifications. 3-Arylsydnones were obtained in 43‒85 % yields. These sydnones are solid with yellow colour and high melting temperature. By Vilsmeier-Haack’s reaction, starting from these sydnones we obtained the corresponding substituted 3-phenyl-4-formylsydnones in 17‒50 % yield (Scheme 1). This reaction has been modified by Shih and Ke’s method [30].

**Scheme 2:**
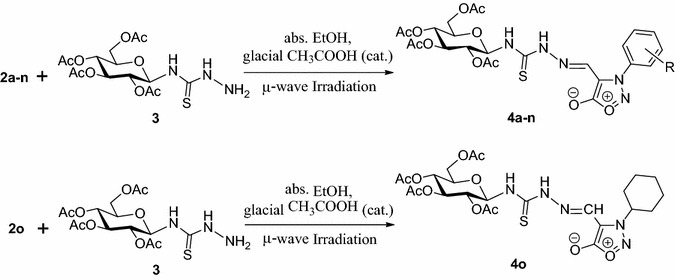
Synthetic pathway for 3-aryl- and 3-cyclohexyl-4-formylsydnone 4-(tetra-*O*-acetyl-β-d-glucopyranosyl)thiosemicarbazones **4a-o**

Condensation reaction of substituted 3-phenyl-4-formylsydnones **2a-o** with *N*-(tetra-*O*-acetyl-β-d-glucopyranosyl)thiosemicarbazide** 3** was carried out on refluxing in the presence of glacial acetic acid as catalyst. These reactions were executed under microwave-assisted heating. All the microwave heating experiments were conducted under optimized reaction conditions of power and temperature in reflux-heating conditions that were investigated below (Scheme 2).

It’s known that peracetylated glucopyranosyl thiosemicarbazones, in particular, and thiosemicarbazones containing other sugars, in general, were sometimes synthesized in severe conditions, in the presence of acidic catalysts, such as hydrochloric or acetic acids in organic solvent, such as methanol, ethanol, propanol under conventional heating conditions [10, 13–24]. The reaction time of these protocols are usually lengthy (2‒48 h). Therefore the search for methods of smooth conditions are always laid out. Initially, we prepared a typical peracetylated (β-d-glucopyranosyl)thiosemicarbazone **4a** from 4-formyl-3-phenylsydnone **2a** (R=H) and thiosemicarbazide **3** under the usual conditions in our procedure for synthesis of these thiosemicarbazones (Scheme 2). This procedure used absolute ethanol as solvent, glacial acetic acid as catalyst, and the reaction mixture was heated under conventional heating method or microwave-assisted conditions. We have evaluated the irradiation time and the effect of microwave power on reaction time and product yield for these reactions (Table 1).

**Table 1:** Different microwave powers used for synthesis of **4a** from **2a** and **3** in absolute ethanol

Entry	Microwave power (Watts)	Yield (%)^a,b^
1	800	60
2	600	68
3	450	71
4	300	71
5	100	58
6	Conventional heating	50 (for 2 h)

^a^Catalyst: glacial acetic acid (2 mmol %) in absolute ethanol for 25 min

^b^Isolated yields

In the process of synthesizing the compounds of 3-aryl-4-formylsydnone *N*-(2,3,4,6-tetra-*O*-β-d-glucopyranosyl)thiosemicarbazones **4a**–**o**, the reaction times were monitored by the thin-layer chromatography with eluent system ethyl acetate-toluene (2:1 v/v). In the case of conventional heating method, product was obtained in yield of 50 % for 120 min under refluxing, while in the case of microwave-assisted heating method, this reaction afforded the yield of 71 % in only 25-min irradiation (The reaction time of 25 min was fixed in order to investigate the microwave power). We found that, initially, the pulses of 1 min of microwave irradiation at maximum power (800 W) were applied, but the yields were not reproducible, and it was difficult to maintain the heating of the reaction mixture. On the other hand, the pulses of 1 min allow to monitor when the reaction is complete by TLC, especially, in cases of the compound **4n** which reaction time was 45 min.

The other high microwave power (from 600 to 300 W) were evaluated and the results were similar, except at 450 W the yields were higher (71 %). This higher yield was also achieved at microwave power of 300 W (71 % yield). The influence of irradiation to isolated yield of **4a** was also examined. The results showed that the isolated yields of **4a** were 68, 71, 71.5 and 70 % with irradiation time of 20, 25, 27 and 30 min, respectively. This microwave power (300 W) was chosen as optimized condition, and was applied for synthesis of other thiosemicarbazone **4b**–**o** (Table 2). In the reaction process, products usually separated as colour solid after cooling to room temperature. The structure of 4-aryl-3-formylsydnone *N*-(tetra-*O*-acetyl-β-d-glucopyranosyl)thiosemicarbazones **4a**–**o** were confirmed by spectroscopic methods.

**Table 2:** Synthesis of 3-aryl- and 3-cyclohexyl-4-formylsydnone *N*-(tetra-*O*-acetyl-β-d-glucopyranosyl)thiosemicarbazones (**4a**–**o**) under conventional and μ-wave heating

Entry	R	Reaction time (min)		Yield (%)	
		Conventional heating	MW heating	Conventional heating	MW heating
**4a**	H	100	25	50	71
**4b**	2-Me	120	28	55	75
**4c**	3-Me	130	30	55	73
**4d**	4-Me	130	30	56	76
**4e**	2,3-diMe	130	35	55	70
**4f**	2,4-diMe	130	35	50	68
**4g**	4-Et	120	28	60	83
**4h**	3-OMe	130	30	60	78
**4i**	4-OMe	130	30	60	81
**4j**	4-OEt	130	25	60	82
**4k**	4-F	130	30	55	65
**4l**	4-Br	150	35	55	63
**4m**	4-I	130	35	57	68
**4n**	2-Me-5-Cl	140	45	50	43
**4o**	Cyclohexyl^a^	130	30	60	85

^a^Cyclohexyl group is attached directly to sydnone ring at position 4

We found that, in general, the electronic nature of the substituents R on the benzene ring of 4-arylsydnones does not affect significantly the reaction yields. However, the strong electron-withdrawing substituents such as NO_2_, Cl, Br, I slow down the reaction and prolong reaction time more than the electron-donating groups such as CH_3_, C_2_H_5_, OCH_3_, OC_2_H_5_ (Table 2). The yields of obtained thiosemicarbazones is quite high, from 63 to 85 %, except the compound **4o**, in this case the yield reached only 43 % after 45 min irradiation. As the result, compounds of 3-aryl-4-formylsydnone *N*-(2,3,4,6-tetra-*O*-acetyl-β-d-glucopyranosyl)thiosemicarbazones (**4a**–**o**) have been synthesized with yields of 43‒85 %. Meanwhile, the conventional heating method only gave the yields of 50‒60 % during prolonged reaction time from 100 min to 150 min.

IR spectra show the characteristic absorption bands for two molecular components: sydnone and monosaccharide. IR spectral regions are 3476‒3343 and 3334‒3164 cm^‒1^ (ν_NH thiosemicarbazone_), 1777‒1746 cm^−1^ (ν_C=O_ ester), 1624‒1599 cm^‒1^ (ν_CH=N_), 1228–1222 and 1056–1043 cm^−1^ (ν_COC_ ester), 1092‒1090 cm^‒1^ (ν_C=S_), some bands at 1549–1505 cm^−1^ (ν_C=C_ aromatic). The absorbance of carbonyl-lactone group of the sydnone ring was sometimes superposed partially by carbonyl-ester group in the range 1777‒1746 cm^‒1^. The presence of the characteristic spectral regions for two moieties, 3-arylsydnone and monosaccharide, and characteristic absorbance band in the range 1624‒1600 cm^‒1^ belong to azomethine bond in IR spectra indicated that the reaction of 3-aryl-4-formylsydnones and *N*-(tetra-*O*-acetyl-β-d-glucopyranosyl)thiosemicarbazide was occurred.

**Fig. 1:**
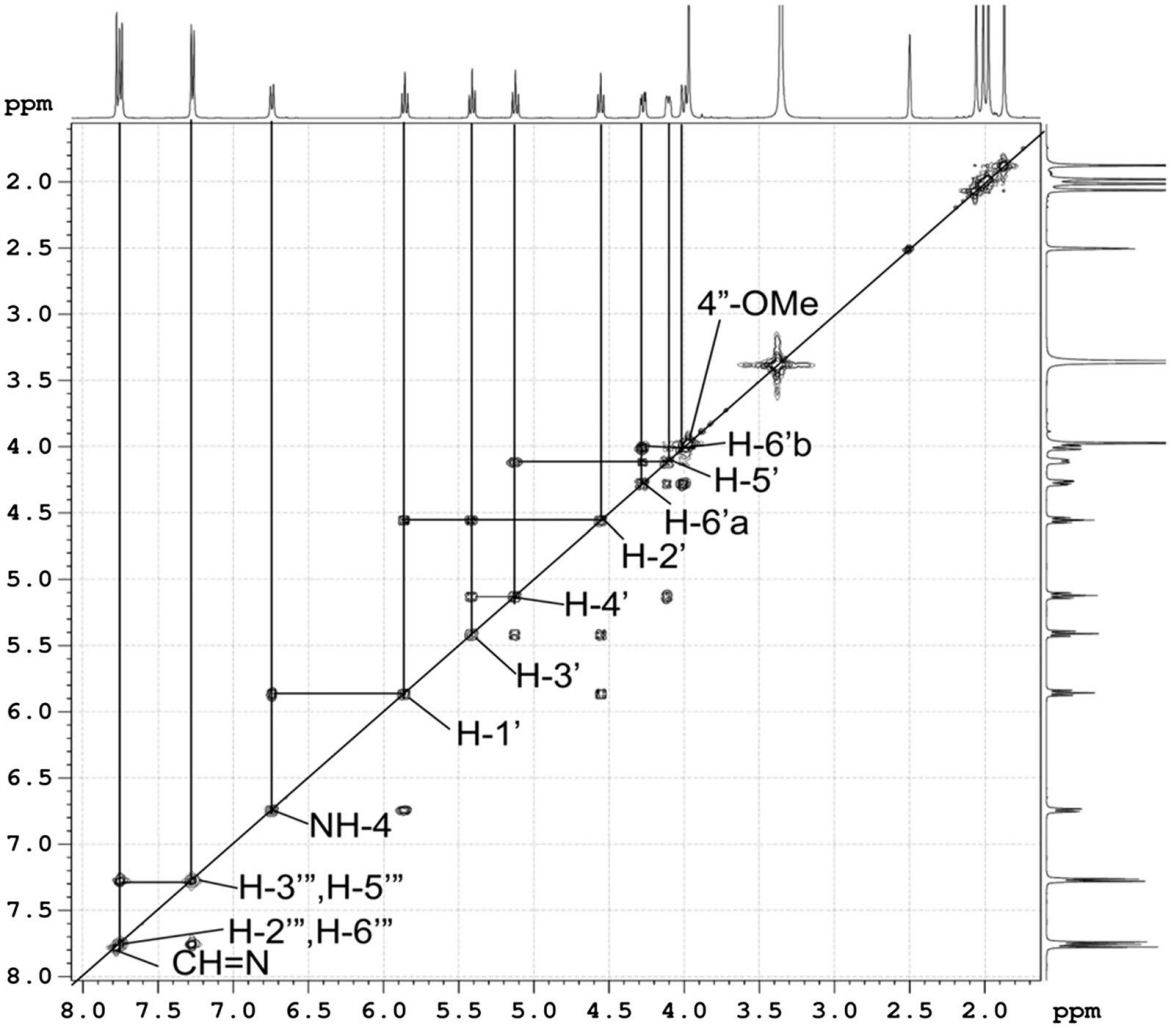
COSY spectrum of thiosemicarbazone **4i**

The ^1^H NMR spectra of these thiosemicarbazones showed the characteristic resonance signals of the protons present in the molecule, which are located in the region of δ = 7.83–6.40 ppm for aromatic protons, δ = 5.87–3.98 ppm for glucopyranose ring. Methyl groups in acetates had signals at δ = 2.07–1.87 ppm. The interaction of protons on neighbour carbons in molecules could be shown in ^1^H–^1^H COSY spectrum of compound **4i** (Fig. 1). The ^13^C NMR spectral data showed the carbon of the aromatic ring with the signals in the δ = 135.5–125.3 ppm, the carbon C-4‴ and C-5‴ of the sydnone ring has characteristic signal is in the range δ = 105.6–104.6 ppm and 165.9‒164.6 ppm, respectively. The carbon in the glucopyranose had chemical shifts at δ = 81.3–61.2 ppm. Carbon atoms in acetyl groups had signals at δ = 21.5–20.1 ppm (for methyl group) and 170.5–169.2 ppm (for carbonyl group).

**Fig. 2:**
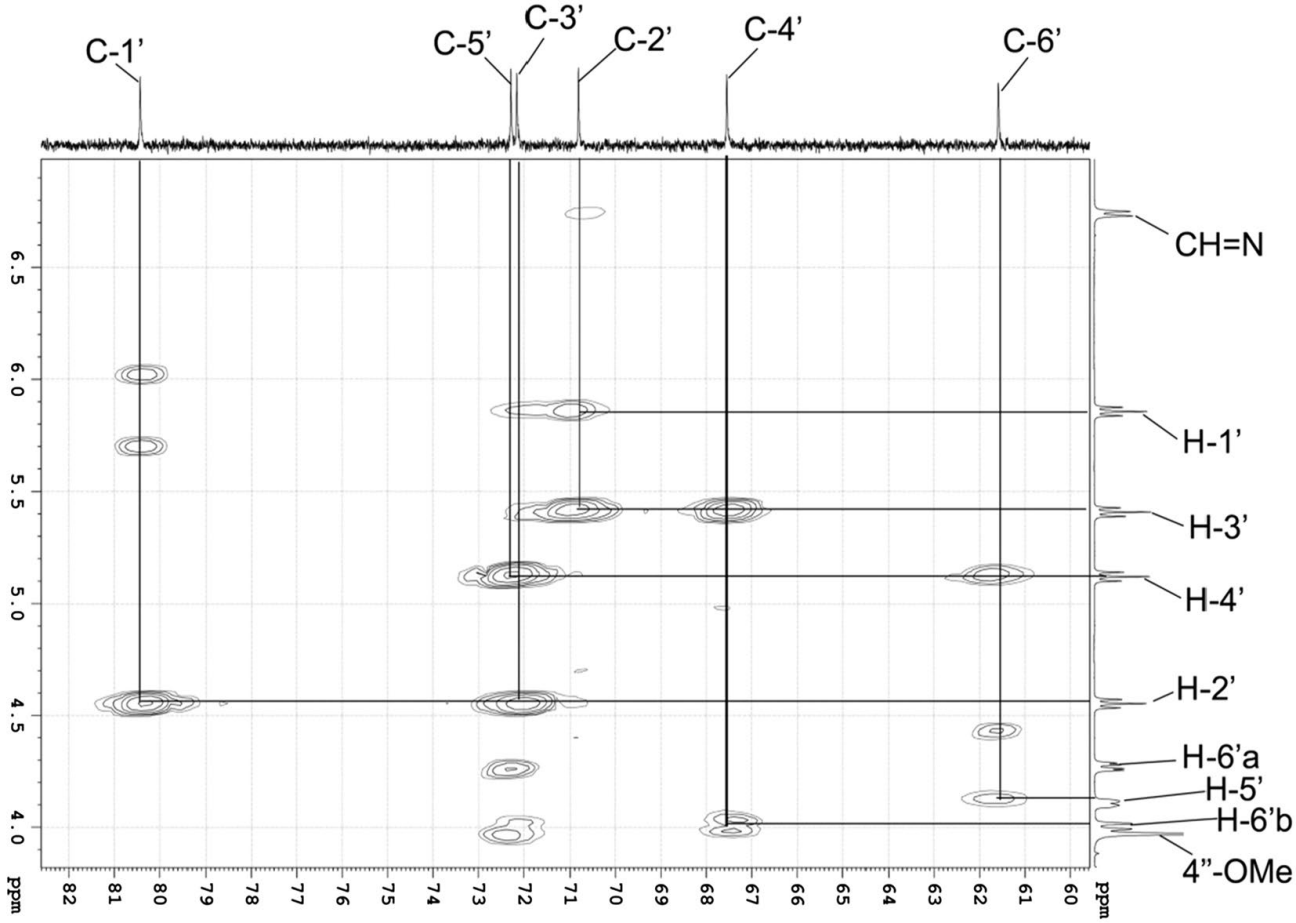
HMBC spectrum of thiosemicarbazone **4i**

From the structure of thiosemicarbazones **4a**–**o** above we can confirm that the presence of sydnone round cannot be used ^1^H NMR spectrum, because the unique C–H bond of sydnone ring substituted by the other group. So the presence of the sydnone ring could be recognized by the presence of resonance signal lying in region at δ = 105.6–104.6 ppm. The HMBC spectral results of compound **4i** showed the long-ranged interaction that appeared in this spectrum (Fig. 2). Some typical ones are below: Carbon atom C-1′ (δ = 80.4 ppm) interacts with proton H-2′ (δ = 4.55 ppm), carbon C-2′ (δ = 70.9 ppm) with protons H-1′ (δ = 5.86 ppm) and H-3′ (δ = 5.41 ppm), carbon C-3′ (δ = 72.1) with protons H-2′ and H-4′ (δ = 5.12 ppm), carbon C-4′ with protons H-3′ and H-6′b (δ = 4.00 ppm).

### Antimicrobial screening

#### Antibacterial activities

Bacterium *Staphylococcus epidermidis* an cause a range of illnesses, from minor skin infections, such as pimples, impetigo, boils (furuncles), cellulitis folliculitis, carbuncles, scalded skin syndrome, and abscesses, to life-threatening diseases such as pneumonia, meningitis, osteomyelitis, endocarditis, toxic shock syndrome (TSS), bacteremia,… It is not a known human pathogen or disease causing agent. *Bacillus subtilis* produces the enzyme subtilisin, which has been reported to cause dermal allergic or hypersensitivity reactions in individuals repeatedly exposed to this enzyme. The bacteria *Salmonella* is commonly associated with food poisoning in countries all over the world, and the species that most people refer to when they talk about *Salmonella* is *S. enterica*. *Salmonella* infections can originate from household pets containing the bacteria, particularly reptiles, improperly prepared meats and seafood, or the surfaces of raw eggs, fruits, or vegetables that have not been adequately disinfected. As their name suggests *Salmonella enterica* are involved in causing diseases of the intestines (enteric means pertaining to the intestine). The three main serovars of *Salmonella enterica* are Typhimurium, Enteritidis, and Typhi.

The ability of thiosemicarbazones **4a**–**o** to inhibit the bacterial growth were screened in vitro at 500 μg/mL concentration against *Staphylococcus epidermidis* and *Bacillus subtilis* as Gram positive bacteria, *Escherichia coli* and *Pseudomonas aeroginosa* as Gram negative bacteria using ciprofloxacin as standard antibacterial reference. The obtained results of testing antimicrobial activities of 3-aryl-4-formylsydnone *N*-(2,3,4,6-tetra-*O*-β-d-glucopyranosyl)thiosemicarbazones **4a**–**o** shows that some substances have significant bacterial inhibitory effects, but are less active than ciprofloxacin. The data from Table 3 revealed that almost all thiosemicarbazones have insignificant activity against *Staphylococcus epidermidis* except compounds **4i**, **4m** and **4n** that medium one. Almost all compounds are remarkable active to *Bacillus subtilis* except thiosemicarbazones **4b**, **4c**, **4g**, and **4h**. In general, thiosemicarbazone **4a**–**o** are more active to Gram negative bacteria, namely *Escherichia coli* and *Salmonella enterica* (Table 3), except compounds **4j** and **4o**.

**Table 3:** Antibacterial activity (paper disc diffusion method) of thiosemicarbazones **4a**–**o**

Entry	Gram positive bacteria		Gram negative bacteria	
	*S*. *epidermidis*	*B. subtilis*	*E. coli*	*S. enterica*
**4a**	14	25	26	27
**4b**	13	16	25	26
**4c**	14	17	26	27
**4d**	14	27	28	31
**4e**	13	28	28	29
**4f**	14	27	29	30
**4g**	14	19	30	31
**4h**	13	20	29	30
**4i**	20	27	31	32
**4j**	14	28	14	13
**4k**	14	32	32	33
**4l**	14	34	34	33
**4m**	24	34	34	35
**4n**	19	32	31	30
**4o**	14	25	13	14
Ciprofloxacin	43	44	42	45
Control	‒	‒	‒	‒

Zone diameter of growth inhibition (mm) after 24 h: 50 μL of stock solution was applied in each hole of each paper disk, i.e. 25 μg/hole. Ciprofloxacin is used as a standard antibacterial reference. Control sample is 10 % DMSO solution in water

The MIC data in Table 4 indicated that almost all the compounds **4a**–**o** showed good antibacterial activity, and some of them had the one similar to the standard drug ciprofloxacin, determined through the serial tube dilution method. Thiosemicarbazone **4k**–**n** were more active against *S*. *epidermidis* than other ones with MIC values of 0.156 μg/mL. All compounds showed significant activities for all bacterial strains used. Among these thiosemicarbazones, compounds **4k**, **4l**, **4m** and **4n** were more active against all tested bacterial strains, especially against *S*. *epidermidis*, *B. subtilis* and *E. coli*. The MIC values in these cases are 0.156, 0.156 and 0.313 μg/mL, respectively. Compounds **4k**, **4l**, **4m** and **4n** contain fluorine, bromine, iodine and chlorine group, respectively, whereas the remained thiosemicarbazones contains no halogen group in benzene ring. Overall most of the compounds exhibit excellent antibacterial activity against the both tested Gram positive and Gram negative bacteria as compared to standard drug ciprofloxacin.

**Table 4:** Antibacterial activity (minimum inhibitory concentration, μg/mL) of thiosemicarbazones **4a**–**o**

Entry	Gram positive bacteria		Gram negative bacteria	
	*S*. *epidermidis*	*B. subtilis*	*E. coli*	*S. enterica*
**4a**	0.313	0.313	0.313	0.625
**4b**	0.313	0.313	0.625	0.313
**4c**	0.313	0.625	0.313	0.313
**4d**	0.313	0.313	0.313	0.625
**4e**	0.313	0.313	0.625	0.625
**4f**	0.313	0.625	0.313	0.625
**4g**	0.313	0.313	0.313	0.313
**4h**	0.313	0.313	0.313	0.625
**4i**	0.625	0.313	0.313	0.625
**4j**	0.313	0.313	0.313	0.625
**4k**	0.156	0.313	0.156	0.313
**4l**	0.156	0.156	0.156	0.313
**4m**	0.156	0.156	0.156	0.313
**4n**	0.156	0.156	0.156	0.313
**4o**	0.313	0.313	0.313	0.625
Ciprofloxacin	0.078	0.156	0.078	0.156
Control	‒	‒	‒	‒

#### Antifungal activities

There are over 20 species of *Candida* yeasts that can cause infection in humans, the most common of which is *Candida albicans*. *Candida* yeasts normally live on the skin and mucous membranes without causing infection; however, overgrowth of these organisms can cause symptoms to develop. Symptoms of candidiasis vary depending on the area of the body that is infected. Fungus *Fusarium oxysporum* plays the role of a silent assassin—the pathogenic strains of this fungus can be dormant for 30 years before resuming virulence and infecting a plant. *F. oxysporum* is infamous for causing a condition called Fusarium wilt. Furthermore, *F. oxysporum* can be harmful to both humans and animals, with its mycotoxins causing the diseases fungal keratitis, Onychomycosis, and Hyalohyphomycosis. *Aspergillus niger* is a fungus and one of the most common species of the genus *Aspergillus*. It causes a disease called black mould on certain fruits and vegetables such as grapes, apricots, onions, and peanuts, and is a common contaminant of food, but may also infect humans through inhalation of fungal spores.

The thiosemicarbazones **4a**–**o** were screened against three fungal strains, namely *Candidaalbicans*, *Fusarium oxysporum* and *Aspergillus niger*. Tested concentration of these thiosemicarbazones is 500 μg/mL using nystatin as standard antifungal reference. Almost all tested compounds have remarkable activities against these three fungal strains, but are less active than nystatin (Table 5). All compounds are significantly active to two first fungi, except substances **4b**, **4c**, **4g**, **4h** (against *C. albicans*) and **4j**, **4o** (against *F. oxysporum*). Almost all thiosemicarbazones are resistant to fungus *A. niger*, except compound **4j**.

**Table 5:** Antifungal activity (paper disc diffusion method) of thiosemicarbazones **4a**–**o**

**Entry**	*C. albicans*	*F. oxysporum*	*A. niger*
**4a**	24	26	14
**4b**	16	27	13
**4c**	18	25	14
**4d**	26	26	23
**4e**	25	25	14
**4f**	25	25	13
**4g**	22	26	24
**4h**	21	25	22
**4i**	25	28	24
**4j**	27	14	26
**4k**	33	32	24
**4l**	34	35	14
**4m**	35	34	23
**4n**	31	30	24
**4o**	26	14	14
Nystatin	44	45	43
Control	‒	‒	‒

Zone diameter of growth inhibition (mm) after 24 h: 50 μL of stock solution was applied in each hole of each paper disk, i.e. 25 μg/hole. Nystatin is used as a standard antifungal reference. Control sample is 10 % DMSO solution in water

The MIC values listed in Table 6 showed that all thiosemicarbazones had good antibacterial activity, but almost all compounds were equal or less active than the standard drug nystatin, determined through the serial tube dilution method. All compounds showed weak to moderate antifungal activity against *C. albicans* and *A. niger* than nystatin (MIC = 0.156‒0.625 μg/mL *vs.* MIC = 0.078 μg/mL of nystatin), and thiosemicarbazones **4l**, **4m** and **4n** exhibited significant activity with MIC = 0.156 μg/mL. These compounds also had good antifungal activity against *F. oxysporum* similarly to nystatin (MIC = 0.156 μg/mL). Among the tested compounds having halogen group **4k**, **4l**, **4m** and **4n** showed highest activity against three strains of fungal organisms.

**Table 6:** Antifungal activity (minimum inhibitory concentration, μg/mL) of thiosemicarbazones **4a**–**o**

Entry	*C. albicans*	*F. oxysporum*	*A. niger*
**4a**	0.625	0.313	0.625
**4b**	0.313	0.625	0.313
**4c**	0.313	0.156	0.313
**4d**	0.313	0.156	0.625
**4e**	0.625	0.625	0.625
**4f**	0.625	0.625	0.625
**4g**	0.313	0.313	0.156
**4h**	0.313	0.313	0.156
**4i**	0.313	0.313	0.625
**4j**	0.625	0.313	0.625
**4k**	0.313	0.156	0.156
**4l**	0.156	0.156	0.156
**4m**	0.156	0.156	0.156
**4n**	0.156	0.156	0.156
**4o**	0.313	0.313	0.625
Nystatin	0.078	0.078	0.156
Control	–	‒	‒

## Conclusions

The authors have developed an effective method for synthesis of 4-formyl-3-arylsydnone *N*-(2,3,4,6-tetra-*O*-acetyl-β-d-glucopyranosyl)thiosemicarbazones under microwave-assisted conditions. These thiosemicarbazones have been obtained in good to excellent yields, except compound **4o**, and fully characterized on the basis of their detailed spectral studies. Among the tested compounds having halogen group **4k**, **4l**, **4m** and **4n** showed highest activity against all tested strains of bacterial and fungal organisms. This heating method is advantageous in having a smaller solvent volume and a shorter reaction time. We also believe that the procedural simplicity, the efficiency and the easy accessibility of the reaction components give access to a wide array of heterocyclic frameworks bearing monosaccharide moiety. Almost all synthesized compounds had their antibacterial and antifungal activities evaluated and showed remarkable results. In summary, we have developed a clean and efficient methodology for the synthesis of novel thiosemicarbazone derivatives bearing sydnone ring and d-glucose moiety; the heterocyclic and monosaccharide system being connected via ‒NH‒C(=S)NH‒N=C< linker using molecular modification approach. The methodology could be further extended and used for the synthesis of other thiosemicarbazones of biological importance.

## Experimental section

### General methods

All chemicals used for the synthesis of the desired compounds were obtained from Merck chemicals. All other commercial reagents were used as received without additional purification. Melting points were measured on STUART SMP3 (BIBBY STERILIN, UK). The FTIS-spectra was recorded on Impact 410 FT-IR Spectrometer (Nicolet, USA), as KBr discs. The ^1^H NMR and ^13^C NMR spectra were recorded on an Avance Spectrometer AV500 (Bruker, Germany) at 500.13 and 125.77 MHz, respectively, using DMSO-*d*_*6*_ as solvent and TMS as an internal standard. Mass spectra were recorded on mass spectrometer LC–MS LTQ Orbitrap XL (ThermoScientific, USA) or Agilent 6310 Ion Trap (Agilent Technologies, USA) in methanol, using ESI method. Thin-layer chromatography was performed on silica gel plates 60F_254_ No. 5715 (Merck, Germany) with toluene: ethyl acetate = 1:2 (by volume) as solvent system, and spots were visualized with UV light or iodine vapour. *N*-(Tetra-*O*-acetyl-β-d-glucopyranosyl)thiosemicarbazide was synthesized using the method which described in Ref. [24] from corresponding isothiocyanate. Tetra-*O*-acetyl-β-glucopyranosyl isothiocyanate were prepared by the reaction of tetra-*O*-acetyl-β-glucopyranosyl bromide with dry ammonium thiocyanate in absolute acetonitrile using tetrabutylammonium bromide as transfer catalyst (modifying the Tashpulatov’s method [19, 20]). This bromide derivative was prepared from d-glucose using Lemieux’s procedure [31]. The obtained thiosemicarbazones were yellow or orange solids, insoluble in water, but easily soluble in ethanol, methanol, benzene, dichloromethane, chloroform, ethyl acetate.

### Synthesis of *N*-(tetra-*O*-acetyl-β-d-glucopyranosyl)thiosemicarbazide (3)

To a solution of 2,3,4,6-tetra-*O*-acetyl-β-d-glucopyranosyl isothiocyanate (3.89 g, 10 mmol) in 25 mL of absolute ethanol, a solution of 85 % hydrazine hydrate (10 mmol, 1.2 ml) in 10 mL of absolute ethanol was added dropwise slowly with stirring in 30 min so that the reaction temperature is below 10 °C. The white precipitate appears immediately when several drops of hydrazine are added due to low solubility of this thiosemicarbazide in ethanol. The temperature of solution was maintained between 10 and 12 °C. The mixture was continuously stirred at 20 °C for 30 min. The solid product then was isolated by filtering with suction. The crude product was crystallized from 96 % ethanol to yield 3.75 g of white product **3**. Yield 85 %, mp 156–158 °C; Ref. [19]: 169‒171 °C. IR (KBr, cm^‒1^): ν 3322, 3129 (ν_NH_), 1752 (ν_C=O ester_), 1355 (ν_C=S_), 1242, 1043 (ν_COC ester_); ^1^H NMR (DMSO-*d*_6_) δ (ppm): 12.77 (s, 1H, NH_b_), 9.23 (s, 1H, NH), 8.17 (s, 1H, NH), 4.58 (s, 2H, NH_2_), 5.80 (m, 1H, H-1), 5.07 (t, *J* = 9.5 Hz, 1H, H-2), 5.34 (t, *J* = 9.75 Hz, 1H, H-3), 4.91 (t, *J* = 9.75 Hz, 1H, H-4), 4.14 (dd, *J* = 12.25, 4.75 Hz, 1H, H-6a), 3.98‒3.93 (m, 2H, H-5 & H-6b), 1.98–1.94 (s, 12H, 4 × *CH*_*3*_CO); ^13^C NMR (DMSO-*d*_6_) δ (ppm): 182.1 (C=S), 169.9–169.2 (4 × *CO*CH_3_), 81.0 (C-1), 70.5 (C-2), 72.5 (C-3), 68.1 (C-4), 72.1 (C-5), 61.8 (C-6), 20.4–20.2 (4 × *CH*_*3*_ CO); MS (+ESI): *m/z* (%) = 422.42 (45) [M+H]^+^, 462.28 (100) [M+K]^+^; calcd. for C_15_H_23_N_3_O_9_S = 421.12 Da.

### General procedure for synthesis of 3-aryl-4-formylsydnone *N*-(tetra-*O*-acetyl-β-d-glucopyranosyl)thiosemicarbazones (4a-o)

To a solution of *N*-(tetra**-***O***-**acetyl-β-d-glucopyranosyl)thiosemicarbazide **3** (2 mmol) in absolute ethanol (5 mL) was added substituted 3-aryl-4-formylsydnone **2a**–**o** (2 mmol). Glacial acetic acid (2 mmol%) as catalyst was added dropwise with stirring. The obtained mixture was then irradiated in microwave oven for 25‒45 min (Tables 1, 2), cooled to room temperature, the separated precipitate was filtered and recrystallized from 96 % ethanol to afford **4a**–**o.**

### 3-Phenyl-4-formylsydnone *N*-(2,3,4,6-tetra-*O*-acetyl-β-d-glucopyranosyl)thiosemicarbazone (**4a**)

Pale yellow crystals, mp 137‒138 °C (from 96 % ethanol), R_f_ = 0.57; $[\alpha ]_{\text{D}}^{25}$ +44.0 (*c* = 0.21, CHCl_3_); FTIR (KBr): ν/cm^‒1^ 3343, 3122 (ν_NH_), 1750 (ν_C=O_ ester and sydnone), 1600 (ν_CH=N_), 1541 (ν_C=C_), 1080 (ν_C=S_), 1235, 1037 (ν_COC_ ester); ^1^H NMR (500 MHz, DMSO-*d*_6_): δ 12.96 (s, 1H, NH-2), 7.83‒7.74 (m, 5H, H-2‴, H-3‴, H-4‴, H-5‴, H-6‴), 7.79 (s, 1H, CH=N), 7.05 (d, 1H, *J* = 9.5 Hz, NH-4), 5.88 (t, 1H, *J* = 9.5 Hz, H-1ʹ), 5.40 (t, 1H, *J* = 9.5 Hz, H-3ʹ), 5.02 (t, 1H, *J* = 9.75 Hz, H-4ʹ), 4.81 (t, 1H, *J* = 9.5 Hz, H-2ʹ), 4.23 (dd, 1H, *J* = 4.5, 12.25 Hz, H-6ʹa), 4.09 (ddd, 1H, *J* = 1.75, 3.75, 9.75 Hz, H-5ʹ), 3.99 (dd, 1H, *J* = 1.0, 12.25 Hz, H-6ʹb), 2.06‒1.90 (s, 12H, 4 × CH_3_CO); ^13^C NMR (125 MHz, DMSO-*d*_6_): δ 177.7 (C=S), 170.5‒169.8 (4 × CH_3_*CO*), 165.6 (C-5ʹʹ), 134.4 (C-1‴), 132.8 (C-3‴, C-4‴, C-5‴), 130.1 (CH = N), 126.0 (C-2‴, C-6‴), 105.6 (C-4ʹʹ), 81.3 (C-1ʹ), 72.9 (C-3ʹ), 72.7 (C-5ʹ), 71.3 (C-2ʹ), 68.3 (C-4ʹ), 61.2 (C-6ʹ), 21.0‒20.6 (4 × *CH*_*3*_CO); ESI–MS (+MS): *m/z* (%) 594.01 (M + H, 67), 407.12 (25), 390.21 (10), 348.17 (20), 331.28 (8), 218.28 (5), 190.37 (8), 176.39 (60), 132,56 (7), 117.41 (100), 102.78 (60), 76.75 (10), 74.59 (33), 59.47 (55); calc. for C_24_H_27_N_5_O_11_S = 593.14 Da.

### 3-(2-Methylphenyl)-4-formylsydnone *N*-(2,3,4,6-tetra-*O*-acetyl-β-d-glucopyranosyl)thiosemicarbazone (**4b**)

Pale yellow crystals, mp 119‒121 °C (from 96 % ethanol), R_f_ = 0.60; $[\alpha ]_{\text{D}}^{25}$ +47.0 (*c* = 0.22, CHCl_3_); FTIR (KBr): ν/cm^‒1^ 3343 (ν_NH_), 1749 (ν_C=O_ ester and sydnone), 1600 (ν_CH=N_), 1521 (ν_C=C_), 1051 (ν_C=S_), 1222, 1056 (ν_COC_ ester); ^1^H NMR (500 MHz, DMSO-*d*_6_): δ 12.0 (s, 1H, NH-2), 7.72 (s, 1H, CH = N), 7.71–7.68 (m, 2H, NH-4, H-3‴), 7.65‒7.60 (m, 1H, H-5‴), 7.60‒7.50 (m, 1H, H-4‴), 6.50‒6.40 (m, 1H, H-6‴), 5.85 (t, 1H, *J* = 9.5 Hz, H-1ʹ), 5.40 (t, 1H, *J* = 9.5 Hz, H-3ʹ), 5.05 (t, 1H, *J* = 10.0 Hz, H-4ʹ), 4.75 (t, 1H, *J* = 9.5 Hz, H-2ʹ), 4.26 (dd, 1H, *J* = 4.5, 12.0 Hz, H-6ʹa), 4.10 (ddd, 1H, *J* = 2.0, 4.0, 10.0 Hz, H-5ʹ), 3.99 (d, 1H, *J* = 12.0 Hz, H-6ʹb), 2.21 (s, 3H, 2‴-CH_3_), 2.09‒1.90 (s, 12H, 4 × CH_3_CO); ^13^C NMR (125 MHz, DMSO-*d*_6_): δ 176.9 (C=S), 170.0‒169.3 (4 × CH_3_*CO*), 165.5 (C-5ʹʹ), 133.6 (C-1‴), 132.3 (C-3‴), 131.6 (C-5‴), 128.8 (C-4‴),128.6 (CH=N), 127.7 (C-6‴), 126.2 (C-2‴), 105.0 (C-4ʹʹ), 80.7 (C-1ʹ), 72.4 (C-5ʹ), 72.2 (C-3ʹ), 70.9 (C-2ʹ), 67.6 (C-4ʹ), 61.7 (C-6ʹ), 20.5‒20.2 (4 × *CH*_*3*_CO), 20.1 (2‴-CH_3_); ESI–MS (‒MS): *m/z* (%) 606.0 (M‒H, 100); calc. for C_25_H_29_N_5_O_11_S = 607.16 Da.

### 3-(3-Methylphenyl)-4-formylsydnone *N*-(2,3,4,6-tetra-*O*-acetyl-β-d-glucopyranosyl)thiosemicarbazone (**4c**)

Yellow crystals, mp 148‒150 °C (from 96 % ethanol), R_f_ = 0.58; $[\alpha ]_{\text{D}}^{25}$ +59.1 (*c* = 0.27, CHCl_3_); FTIR (KBr): ν/cm^‒1^ 3525, 3164 (ν_NH_), 1756 (ν_C=O_ ester and sydnone), 1624 (ν_CH=N_), 1532 (ν_C=C_), 1084 (ν_C=S_), 1237, 1041 (ν_COC_ ester); ^1^H NMR (500 MHz, DMSO-*d*_6_): δ 11.98 (s, 1H, NH-2), 7.78 (s, 1H, CH=N), 7.63‒7.60 (m, 4H, H-2‴, H-4‴, H-5ʹʹ, H-6‴), 7.00 (d, 1H, *J* = 10.0 Hz, NH-4), 5.87 (t, 1H, *J* = 9.5 Hz, H-1ʹ), 5.41 (t, 1H, *J* = 9.5 Hz, H-3ʹ), 5.01 (t, 1H, *J* = 9.75 Hz, H-4ʹ), 4.72 (t, 1H, *J* = 9.5 Hz, H-2ʹ), 4.24 (dd, 1H, *J* = 4.5, 12.5 Hz, H-6ʹa), 4.10 (ddd, 1H, *J* = 2.0, 4.5, 10.0 Hz, H-5ʹ), 3.98 (dd, 1H, *J* = 1.5, 12.0 Hz, H-6ʹb), 2.46 (s, 3H, 3‴-CH_3_), 2.05‒1.90 (s, 12H, 4 × CH_3_CO); ^13^C NMR (125 MHz, DMSO-*d*_6_): δ 177.2 (C=S), 170.0‒169.3 (4 × CH_3_*CO*), 129.5 (CH=N), 80.7 (C-1ʹ), 70.9 (C-2ʹ), 72.2 (C-3ʹ), 67.8 (C-4ʹ), 72.3 (C-5ʹ), 61.7 (C-6ʹ), 104.9 (C-4ʹʹ), 165.1 (C-5ʹʹ), 140.2 (C-1‴), 122.6 (C-2‴), 133.9 (C-3‴), 129.9 (C-4‴), 132.9 (C-5‴), 125.6 (C-6‴), 20.7‒20.16 (4 × *CH*_*3*_CO), 20.7 (3‴-CH_3_); ESI–MS (‒MS): *m/z* (%) 606.1 (M‒H, 100); calc. for C_25_H_29_N_5_O_11_S = 607.16 Da.

### 3-(4-Methylphenyl)-4-formylsydnone *N*-(2,3,4,6-tetra-*O*-acetyl-β-d-glucopyranosyl)thiosemicarbazone (**4d**)

Yellow crystals, mp 149‒151 °C (from 96 % ethanol), R_f_ = 0.58; $[\alpha ]_{\text{D}}^{25}$ +52.3 (*c* = 0.25, CHCl_3_); FTIR (KBr): ν/cm^‒1^ 3329, 3215 (ν_NH_), 1747 (ν_C=O_ ester and sydnone), 1601 (ν_CH=N_), 1510, 1537 (ν_C=C_), 1083 (ν_C=S_), 1226, 1043 (ν_COC_ ester); ^1^H NMR (500 MHz, DMSO-*d*_6_): δ 12.04 (s, 1H, NH-2), 7.70 (s, 1H, CH = N), 7.75 (d, 2H, *J* = 9.0 Hz, H-3‴, H-5‴), 7.27 (d, 2H, *J* = 9.0 Hz, H-2‴, H-6‴), 6.73 (d, 1H, *J* = 10.0 Hz, NH-4), 5.85 (t, 1H, *J* = 9.5 Hz, H-1ʹ), 5.41 (t, 1H, *J* = 9.75 Hz, H-3ʹ), 5.12 (t, 1H, *J* = 9.75 Hz, H-4ʹ), 4.54 (t, 1H, *J* = 9.5 Hz, H-2ʹ), 4.27 (dd, 1H, *J* = 4.5, 12.5 Hz, H-6ʹa), 4.11 (ddd, 1H, *J* = 2.0, 4.5, 10.0 Hz, H-5ʹ), 3.99 (d, 1H, *J* = 12.5 Hz, H-6ʹb), 3.97 (s, 3H, 4‴–CH_3_), 2.06‒1.87 (s, 12H, 4 × CH_3_CO); ^13^C NMR (125 MHz, DMSO-*d*_6_): δ 177.2 (C = S), 170.1‒169.2 (4 × CH_3_*CO*), 165.9 (C-5ʹʹ), 161.5 (C-4‴), 129.9 (CH=N), 126.9 (C-3‴, C-5‴), 126.8 (C-1‴), 115.1 (C-2‴, C-6‴), 104.6 (C-4ʹʹ), 80.4 (C-1ʹ), 72.3 (C-5ʹ), 72.1 (C-3ʹ), 70.8 (C-2ʹ), 67.5 (C-4ʹ), 61.6 (C-6ʹ), 55.8 (4‴-CH_3_), 20.5‒20.1 (4 × *CH*_*3*_CO); ESI–MS (+MS): *m/z* (%) 608.00 (M+H, 55), 536.00 (10), 412.11 (14), 407.15 (20), 390.19 (7), 348.13 (10), 321.36 (25), 290.19 (8), 218.32 (5), 204, 138.30 (55), 139.18 (37), 117.32 (95), 102.45 (100), 81.37 (18), 74.58 (35), 59.45 (55)calc. for C_25_H_29_N_5_O_11_S = 607.16 Da.

### 3-(2,3-Dimethylphenyl)-4-formylsydnone *N*-(2,3,4,6-tetra-*O*-acetyl-β-d-glucopyranosyl)thiosemicarbazone (**4e**)

Pale yellow crystals, mp 138‒140 °C (from 96 % ethanol), R_f_ = 0.53; $[\alpha ]_{\text{D}}^{25}$ +47.0 (*c* = 0.23, CHCl_3_); FTIR (KBr): ν/cm^‒1^ 1750 (ν_C=O_ ester and sydnone), 3338, 3124 (ν_NH_), 1610 (ν_CH=N_), 1490, 1450 (ν_C=C_), 1085 (ν_C=S_), 1039, 1229 (ν_COC_ ester); ^1^H NMR (500 MHz, DMSO-*d*_6_): δ 11.97 (s, 1H, NH-2), 7.70 (s, 1 H, CH = N), 7.39 (t, 2H, *J* = 7.0), H-4‴, H-5‴), 7.61 (s, 1H, H-6‴), 6.33 (dd, 1H, *J* = 9.5 Hz, NH-4), 5.81 (m, 1H, H-1ʹ), 5.36 (t, 2H, *J* = 9.5 Hz, H-3ʹ, H-4ʹ), 4.77 (m, 1H, H-2ʹ), 4.33 (t, 1H, *J* = 11.5 Hz, H-5ʹ), 4.09 (d, 1H, *J* = 9.0 Hz, H-6ʹa, H-6ʹb), 2.45‒2.39 (s, 3H, 2‴-CH_3_), 2.39‒2.09 (s, 12H, 4 × CH_3_CO), 1.89 (s, 3H, 3‴-CH_3_); ^13^C NMR (125 MHz, DMSO-*d*_6_): δ 177.1 (C=S), 170‒169.3 (4 × CH_3_*CO*), 165.6 (C-5ʹʹ), 139.0 (C-1‴), 133.7 (C-2‴), 133.6 (C-3‴), 132.5 (C-4‴), 128.5 (CH=N), 127.1 (C-6‴), 123.7 (C-5‴), 105.1 (C-4ʹʹ), 80.6 (C-1ʹ), 72.1 (C-5ʹ), 71.7 (C-3ʹ), 71.4 (C-2ʹ), 67.6 (C-4ʹ), 61.6 (C-6ʹ), 20.5‒20.1 (4 × *CH*_*3*_CO), 13.2 (2‴-CH_3_), 19.7 (3‴-CH_3_); ESI–MS (+MS): *m/z* (%) 622.03 (M+H, 87), 600.44 (5), 590.29 (10), 556.47 (8), 473.51 (10), 407.29 (10), 390.41 (6), 348.25 (12), 331.40 (6), 218.39 (12), 202.42 (40), 132.44 (8), 122.33 (10), 117.36 (100), 102.59 (38), 74.43 (25), 59.18 (53); calc. for C_26_H_31_N_5_O_11_S = 621.17 Da.

### 3-(2,4-Dimethylphenyl)-4-formylsydnone *N*-(2,3,4,6-tetra-*O*-acetyl-β-d-glucopyranosyl)thiosemicarbazone (**4f**)

Pale yellow crystals, mp 119‒121 °C (from 96 % ethanol), R_f_ = 0.55; $[\alpha ]_{\text{D}}^{25}$ +46.0 (*c* = 0.22, CHCl_3_); FTIR (KBr): ν/cm^‒1^ 1753 (ν_C=O_ ester and sydnone), 3334, 3256 (ν_NH_), 1600 (ν_CH=N_), 1530, 1450 (ν_C=C_), 1080 (ν_C=S_), 1039, 1224 (ν_COC_ ester); ^1^H NMR (500 MHz, DMSO-*d*_6_): δ 12.04 (s, 1H, NH-2), 7.74 s, 1H, CH=N), 7.57 (t, 1H, *J* = 8.0 Hz, H-3‴), 7.42 (s, 1H, H-6‴), 7.35 (t, 1H, *J* = 8.0 Hz, H-5‴), 6.57 d; 1H, *J* = 10.0 Hz, NH-4), 5.89 (m, 1H, H-1ʹ), 5.42 (m, 1H, H-3ʹ), 5.05 (s, 1H, H-4ʹ), 4.62 (s, 1H, H-2ʹ), 4.21 (m, 1H, H-5ʹ), 4.15 d; 1H, *J* = 10.0 Hz, H-6ʹa), 3.99 d; 1H, *J* = 5.75 Hz, H-6ʹb), 2.01‒1.90 (s, 12 H, 4 × *CH*_*3*_CO), 2.52 (s, 3H (2‴–CH_3_), 2.12 (s, 3H (4‴–CH_3_); ^13^C NMR (125 MHz, DMSO-*d*_6_): δ 177.2 (C=S), 169.9‒169.2 (4 × CH_3_*CO*), 165.6 (C-5ʹʹ), 142.0 (C-1‴), 133.4 (C-4‴), 131.9 (C-2‴), 131.2 (C-5‴), 129.1 (CH=N), 127.9 (C-3‴), 126.0 (C-6‴), 104.9 (C-4ʹʹ), 80.6 (C-1ʹ), 72.5 (C-5ʹ), 70.9 (C-3ʹ), 67.8 (C-2ʹ), 65.0 (C-6ʹ), 61.6 (C-4ʹ), 20.7‒20.1 (4 × *CH*_*3*_CO), 21.0 (4‴-CH_3_), 16.1 (2‴-CH_3_); ESI–MS (+MS): *m/z* (%) 622.07 (M + H, 100), 607.11 (10), 331.29 (6), 315.32 (20), 277.08 (5), 247.60 (50), 219.29 (13), 189.51 (14), 161.50 (6), 132.50 (15), 117.25 (85), 102.56 (10), 74.29 (6), 58.12 (47); calc. for C_26_H_31_N_5_O_11_S = 621.17 Da.

### 3-(4-Ethylphenyl)-4-formylsydnone *N*-(2,3,4,6-tetra-*O*-acetyl-β-d-glucopyranosyl)thiosemicarbazone (**4g**)

Pale yellow crystals, mp 138‒140 °C (from 96 % ethanol), R_f_ = 0.58; $[\alpha ]_{\text{D}}^{25}$ +59.0 (*c* = 0.27, CHCl_3_); FTIR (KBr): ν/cm^‒1^ 3310, 3228 (ν_NH_), 1777 (ν_C=O_ ester and sydnone), 1600 (ν_CH=N_), 1551, 1518 (ν_C=C_), 1084 (ν_C=S_), 1228, 1043 (ν_COC_ ester); ^1^H NMR (500 MHz, DMSO-*d*_6_): δ 12.01 (s, 1H, NH-2), 7.81 (s, 1H, CH = N), 7.74 (d, 2H, *J* = 8.25 Hz, H-3‴, H-5‴), 7.58 (d, 2H, *J* = 8.25 Hz, H-2‴, H-6‴), 7.08 (d, 1H, *J* = 10.0 Hz, NH-4), 5.90 (t, 1H, *J* = 9.5 Hz, H-1ʹ), 5.44 (t, 1H, *J* = 9.5 Hz, H-3ʹ), 5.00 (t, 1H, *J* = 9.5 Hz, H-4ʹ), 4.73 (t, 1H, *J* = 9.5 Hz, H-2ʹ), 4.19 (dd, 1H, *J* = 4.5, 12.5 Hz, H-6ʹa), 4.10 (ddd, 1H, *J* = 2.0, 4.5, 10.0 Hz, H-5ʹ), 3.99 (dd, 1H, *J* = 1.5, 12.5 Hz, H-6ʹb), 2.85 (q, 2H, *J* = 7.5 Hz, 4‴-*CH*_*2*_CH_3_), 2.04‒1.91 (s, 12H, 4 × *CH*_*3*_CO), 1.30 (t, 3H, *J* = 7.5 Hz, 4‴-CH_2_*CH*_*3*_); ^13^C NMR (125 MHz, DMSO-*d*_6_): δ 177.3 (C=S), 170.0‒169.3 (4 × CH_3_*CO*), 165.2 (C-5ʹʹ), 148.5 (C-1‴), 131.6 (C-4‴), 129.9 (CH=N), 129.1 (C-3‴, C-5‴), 125.4 (C-2‴, C-6‴), 104.8 (C-4ʹʹ), 80.7 (C-1ʹ), 72.3 (C-5ʹ), 72.1 (C-3ʹ), 70.9 (C-2ʹ), 67.7 (C-4ʹ), 61.4 (C-6ʹ), 28.0 (4‴-*CH*_*2*_CH_3_), 20.6‒20.2 (4 × *CH*_*3*_CO), 15.0 (4‴-CH_2_*CH*_*3*_); ESI–MS (‒MS): *m/z* (%) 620.3 (M‒H, 100); calc. for C_26_H_31_N_5_O_11_S = 621.17 Da.

### 3-(3-Methoxyphenyl)-4-formylsydnone *N*-(2,3,4,6-tetra-*O*-acetyl-β-d-glucopyranosyl)thiosemicarbazone (**4h**)

Yellow crystals, mp 139‒141 °C (from 96 % ethanol), R_f_ = 0.60; $[\alpha ]_{\text{D}}^{25}$ +53.2 (*c* = 0.24, CHCl_3_); FTIR (KBr): ν/cm^‒1^ 3476, 3334 (ν_NH_), 1756 (ν_C=O_ ester and sydnone), 1609 (ν_CH=N_), 1528 (ν_C=C_), 1093 (ν_C=S_), 1228, 1040 (ν_COC_ ester); ^1^H NMR (500 MHz, DMSO-*d*_6_): δ 11.97 (s, 1H, NH-2), 7.81 (s, 1H, CH=N), 7.64 (t, 1H, *J* = 7.5 Hz, H-5‴), 7.47 (t, 1H, *J* = 2.0 Hz, H-2‴), 7.38 (dd, 1H, *J* = 1.0, 7.5 Hz, H-4‴), 7.34 (dd, 1H, *J* = 2.0, 7.5 Hz, H-6‴), 7.18 (d, 1H, *J* = 9.5 Hz, NH-4), 5.88 (t, 1H, *J* = 9.5 Hz, H-1ʹ), 5.42 (t, 1H, *J* = 9.5 Hz, H-3ʹ), 5.00 (t, 1H, *J* = 9.5 Hz, H-4ʹ), 4.80 (t, 1H, *J* = 9.5 Hz, H-2ʹ), 4.21 (dd, 1H, *J* = 5.0, 12.25 Hz, H-6ʹa), 4.10 (ddd, 1H, *J* = 2.0, 4.5, 10.0 Hz, H-5ʹ), 3.99 (dd, 1H, *J* = 1.5, 12.25 Hz, H-6ʹb), 3.86 (s, 3H, 3‴-OCH_3_), 2.05‒1.90 (s, 12H, 4 × *CH*_*3*_CO); ^13^C NMR (125 MHz, DMSO-*d*_6_): δ 177.2 (C=S), 170.1‒169.3 (4 × CH_3_*CO*), 164.8 (C-5ʹʹ), 160.0 (C-3‴), 134.8 (C-1‴), 131.0 (C-5‴), 129.7 (CH = N), 118.4 (C-6‴), 117.5 (C-4‴), 111.0 (C-2‴), 105.1 (C-4ʹʹ), 80.8 (C-1ʹ), 72.3 (C-5ʹ), 72.2 (C-3ʹ), 71.0 (C-2ʹ), 67.9 (C-4ʹ), 61.8 (C-6ʹ), 55.8 (3‴-OCH_3_), 20.5‒20.2 (4 × *CH*_*3*_CO); ESI–MS (‒MS): *m/z* (%) 622.3 (M‒H, 100); calc. for C_25_H_29_N_5_O_12_S = 623.15 Da.

### 3-(4-Methoxyphenyl)-4-formylsydnone *N*-(2,3,4,6-tetra-*O*-acetyl-β-d-glucopyranosyl)thiosemicarbazone (**4i**)

Light yellow crystals, mp 160‒162 °C (from 96 % ethanol), R_f_ = 0.58; $[\alpha ]_{\text{D}}^{25}$ +65.0 (*c* = 0.26, CHCl_3_); FTIR (KBr): ν/cm^‒1^ 3344, 3260 (ν_NH_), 1746 (ν_C=O_ ester and sydnone), 1599 (ν_CH=N_), 1549, 1505 (ν_C=C_), 1093 (ν_C=S_), 1223, 1043 (ν_COC_ ester); ^1^H NMR (500 MHz, DMSO-*d*_6_): δ 12.02 (s, 1H, NH-2), 7.77 (s, 1H, CH=N), 7.74 (d, 2H, *J* = 8.75 Hz, H-3‴, H-5‴), 7.27 (d, 2H, *J* = 8.75 Hz, H-2‴, H-6‴), 6.75 (d, 1H, *J* = 10.0 Hz, NH-4), 5.86 (t, 1H, *J* = 9.5 Hz, H-1ʹ), 5.41 (t, 1H, *J* = 9.5 Hz, H-3ʹ), 5.12 (t, 1H, *J* = 9.75 Hz, H-4ʹ), 4.55 (t, 1H, *J* = 9.5 Hz, H-2ʹ), 4.27 (dd, 1H, *J* = 4.0, 12.25 Hz, H-6ʹa), 4.12‒4.10 (m, 1H, H-5ʹ), 4.00 (d, 1H, *J* = 12.25 Hz, H-6ʹb), 3.97 (s, 3H, 4‴-OCH_3_), 2.06‒1.78 (s, 12H, 4 × *CH*_*3*_CO); ^13^C NMR (125 MHz, DMSO-*d*_6_): δ 177.2 (C=S), 170.1‒169.3 (4 × CH_3_*CO*), 165.9 (C-5ʹʹ), 161.5 (C-4‴), 129.2 (CH=N), 126.9 (C-1‴), 127.0 (C-3‴, C-5‴), 115.1 (C-2‴, C-6‴), 104.6 (C-4ʹʹ), 80.4 (C-1ʹ), 72.3 (C-5ʹ), 72.1 (C-3ʹ), 70.9 (C-2ʹ), 67.5 (C-4ʹ), 61.6 (C-6ʹ), 55.8 (4‴-OCH_3_), 20.5‒20.1 (4 × *CH*_*3*_CO); ESI–MS (+MS): *m/z*(%) 624.01 (M + H, 100), 556.02 (7), 407.11 (15), 391.21 (5), 348.17 (8), 331.25 (5), 204.21 (75), 124.22 (8), 117.15 (80), 102.25 (95), 84.25 (12), 74.18 (50), 59.08 (67); calc. for C_25_H_29_N_5_O_12_S = 623.15 Da.

### 3-(4-Ethoxyphenyl)-4-formylsydnone *N*-(2,3,4,6-tetra-*O*-acetyl-β-d-glucopyranosyl)thiosemicarbazone (**4j**)

Light yellow crystals, mp 159–161 °C (from 96 % ethanol), R_f_ = 0.60; $[\alpha ]_{\text{D}}^{25}$ +54.0 (*c* = 0.22, CHCl_3_); FTIR (KBr): ν/cm^‒1^ 3324, 3202 (ν_NH_), 1737 (ν_C=O_ ester), 1601 (ν_C=N_), 1548, 1490 (ν_C=C_), 1085 (ν_C=S_), 1234, 1042 (ν_COC_ ester); ^1^H NMR (500 MHz, DMSO-*d*_6_): δ 12.04 (s, 1H, NH-2), 7.78 (s, 1H, CH=N), 7.73 (d, 2H, *J* = 8.75 Hz, H-3‴, H-5‴), 7.24 (d, 2H, *J* = 8.75 Hz, H-2‴, H-6‴), 6.75 (d, 1H, *J* = 10.0 Hz, NH-4), 5.88 (t, 1H, *J* = 9.5 Hz, H-1ʹ), 5.42 (t, 1H, *J* = 9.5 Hz, H-3ʹ), 5.06 (t, 1H, *J* = 9.5 Hz, H-4ʹ), 4.60 (t, 1H, *J* = 9.5 Hz, H-2ʹ), 4.26‒4.18 (m, 1H, H-6ʹa), 4.22 (q, 2H, *J* = 7.5 Hz, 4‴-O*CH*_*2*_CH_3_), 4.10‒4.07 (m, 1H, H-5ʹ), 3.99 (d, 1H, *J* = 12.5 Hz, H-6ʹb), 3.97 (t, 3H, *J* = 7.5 Hz, 4‴-OCH_2_*CH*_*3*_), 2.07‒1.87 (s, 12H, 4 × *CH*_*3*_CO); ^13^C NMR (125 MHz, DMSO-*d*_6_): δ 177.3 (C=S), 170.1‒169.2 (4 × CH_3_*CO*), 165.9 (C-5ʹʹ), 161.5 (C-4‴), 129.3 (CH = N), 126.9 (C-3‴, C-5‴), 126.6 (C-1‴), 115.4 (C-2‴, C-6‴), 104.6 (C-4ʹʹ), 80.5 (C-1ʹ), 72.4 (C-3ʹ), 72.2 (C-5ʹ), 70.7 (C-2ʹ), 67.7 (C-4ʹ), 64.1 (4‴-O*CH*_*2*_CH_3_), 61.6 (C-6ʹ), 20.5‒20.2 (4 × *CH*_*3*_CO), 14.2 (4‴-OCH_2_*CH*_*3*_); ESI–MS (+MS): *m/z*(%) 638.00 (M + H, 60), 432.13 (7), 390.19 (8), 348.11 (10), 331.20 (6), 234.30 (5), 218.29 (45), 190.29 (5), 138.29 (10), 117.27 (100), 102.45 (62), 76.57 (13), 74.45 (23), 59.30 (43); calc. for C_26_H_31_N_5_O_12_S = 637.17 Da.

### 3-(4-Fluorophenyl)-4-formylsydnone N-(2,3,4,6-tetra-*O*-acetyl -β-d-glucopyranosyl)thiosemicarbazon (**4k**)

Light yellow crystals, mp 176‒178 °C (from 96 % ethanol), R_f_ = 0.55; $[\alpha ]_{\text{D}}^{25}$ +47.2 (*c* = 0.24, CHCl_3_); FTIR (KBr): ν/cm^‒1^ 1744 (ν_C=O_ ester and sydnone), 3329, 3186 (ν_NH_), 1597 (ν_CH=N_), 1518, 1550 (ν_C=C_), 1090 (ν_C=S_), 1056, 1229 (ν_COC_ ester); ^1^H NMR (500 MHz, DMSO-*d*_6_): δ 12.00 (s, 1H, NH-2), 7.94‒7.91 (m, 2H, H-3‴,H-5‴), 7.77 (s, 1H, CH=N), 7.58 (t, 2H, *J* = 8.75 Hz, H-2‴, H-6‴), 6.74 (d, 1H, *J* = 10.0 Hz, NH-4), 5.87 (t, 1H, *J* = 9.75 Hz, H-1ʹ), 5.44 (t, 1H, *J* = 9.75 Hz, H-3ʹ), 5.01 (t, 1H, *J* = 9.75 Hz, H-4ʹ), 4.69 (t, 1H, *J* = 9.75 Hz, H-2ʹ), 4.22 (dd, 1H, *J* = 9.0;9.0 Hz, H-5ʹ), 4.10 (m, 1H, H-6ʹa), 4.07‒4.00 (m, 1H, H-6ʹb), 2.05‒1.89 (s, 12H, 4 × *CH*_*3*_CO); ^13^C NMR (125 MHz, DMSO-*d*_6_): δ 177.0 (C=S), 170.7‒169.4 (4 × CH_3_*CO*), 167.2 (C-5ʹʹ), 165.9 (C-4‴), 163.8 (CH=N), 144.1 (C-1‴), 129.9 (C-2‴), 127.6 (C-6‴), 121.8 (C-3‴), 117.0 (C-5‴), 101.3 (C-4ʹʹ), 84.0 (C-1ʹ), 83.9 (C-2ʹ), 73.8 (C-5ʹ), 72.5 (C-3ʹ), 70.4 (C-4ʹ), 61.4 (C-6ʹ), 20.6‒20.5 (4 × *CH*_*3*_CO); ESI–MS (+MS): *m/z* (%) 612.00 (M + H, 100), 580.18 (14), 503.97 (6), 452.18 (5), 391.57 (35), 353.79 (8), 331.25 (8), 296.06 (12), 287.06 (20), 272.29 (25), 246.83 (30), 229.10 (10), 202.44 (25), 189.21 (27), 173.56 (45), 164.51 (14), 144.43 (10), 117.24 (82), 102.27 (53), 84.29 (10), 74.32 (17), 59.20 (53); calc. for C_24_H_26_FN_5_O_11_S = 611.4 Da.

### 3-(4-Bromophenyl)-4-formylsydnone N-(2,3,4,6-tetra-*O*-acetyl-β-d-glucopyranosyl thiosemicarbazon (**4l**)

Dark yellow crystals, mp 157‒159 °C (from 96 % ethanol), R_f_ = 0.53; $[\alpha ]_{\text{D}}^{25}$ +57.3 (*c* = 0.26, CHCl_3_); FTIR (KBr): ν/cm^‒1^ 1746 (ν_C=O_ ester and sydnone), 3083, 3289 (ν_NH_), 1610 (ν_CH=N_), 1478, 1520 (ν_C=C_), 1041 (ν_C=S_), 1036, 1222 (ν_COC_ ester); ^1^H NMR (500 MHz, DMSO-*d*_6_): δ 11.98 (s, 1H, NH-2), 8.05 (d, 2H, *J* = 9.0 Hz, H-3‴, H-45ʹʹ), 7.96 (s, 1H, CH = N), 7.90 (d, 2H, *J* = 8.5 Hz, H-2‴, H-6‴), 6.75 (d, 1H, *J* = 10.0 Hz, NH-4), 5.88 (t, 1H, *J* = 9.5 Hz, H-1ʹ), 5.48 (t, 1H, *J* = 9.5 Hz, H-3ʹ), 5.26 (t, 1H, *J* = 9.75 Hz, H-4ʹ), 4.68 (t, 1H, *J* = 9.5 Hz, H-2ʹ), 4.23 (dd, 1H, *J* = 9.5;8.0 Hz, H-5ʹ), 4.10 (d, 1H, *J* = 10.0 Hz, H-6ʹa), 4.01 (d, 1H, *J* = 12.0 Hz, H-6ʹb), 2.08‒1.89 (s, 12H, 4 × *CH*_*3*_CO); ^13^C NMR (125 MHz, DMSO-*d*_6_): δ 177.4 (C=S), 170.5‒169.8 (4 × CH_3_*CO*), 156.2 (C-5ʹʹ), 136.4 (C-1‴), 133.0 (C-3‴, C-5‴), 128.5 (CH = N), 123.3 (C-2‴,C-6‴), 121.7 (C-4‴), 104.5 (C-4ʹʹ), 81.1 (C-1ʹ), 71.3 (C-2ʹ), 72.9 (C-5ʹ), 72.3 (C-3ʹ), 68.3 (C-4ʹ), 62.2 (C-6ʹ), 21.1‒20.6 (4 × *CH*_*3*_CO); ESI–MS (+MS): calc. for C_24_H_26_^79^BrN_5_O_11_S/C_24_H_26_^81^BrN_5_O_11_S = 671.05/673.05 Da; *m*/*z* (%) 671.13 (100)/673.15 (90) (M^+^), 642.01 (5), 586.32(5), 331.23 (4), 298.36 (5).

### 3-(4-Iodophenyl)-4-formylsydnone *N*-(2,3,4,6-tetra-*O*-acetyl -β-d-glucopyranosyl)thiosemicarbazon (**4m**)

Dark yellow crystals, mp 128‒130 °C (from 96 % ethanol), R_f_ = 0.51; $[\alpha ]_{\text{D}}^{25}$ +55.0 (*c* = 0.20, CHCl_3_); FTIR (KBr): ν/cm^‒1^ 1750 (ν_C=O_ ester and sydnone), 2944, 3355 (ν_NH_), 1521 (ν_CH=N_), 1456, 1521 (ν_C=C_), 1045 (ν_C=S_), 1045, 1226 (ν_COC_ ester); ^1^H NMR (500 MHz, DMSO-*d*_6_): δ 11.99 (s, 1H, NH-2), 8.12 (d, 2H, *J* = 9.0 Hz, H-3‴, H-5‴), 7.80 (s, 1H, CH = N), 7.64 (d, 2H, *J* = 8.5 Hz, H-2‴, H-6‴), 7.06 (d, 1H, *J* = 10.0 Hz, NH-4), 5.91 (t, 1H, 9.5 Hz, H-1ʹ), 5.46 (t, 1H, *J* = 9.75 Hz, H-3ʹ), 5.21 (t, 1H, *J* = 9.75 Hz, H-4ʹ), 4.81 (t, 1H, *J* = 9.5 Hz, H-2ʹ), 4.20 (dd, 1H, *J* = 9.5;9.0 Hz, H-5ʹ), 4.11‒4.07 (m, 1H, H-6ʹa), 4.00 (dd, 1H *J* = 4.0, 3.0 Hz, H-6ʹb), 2.06‒1.90 (s, 12H, 4 × *CH*_*3*_CO); ^13^C NMR (125 MHz, DMSO-*d*_6_): δ 177.3 (C=S), 170.0‒169.2 (4 × CH_3_*CO*), 165.1 (C-5ʹʹ), 138.8 (C-1‴), 132.5 (C-3‴, C-5‴), 129.8 (CH=N), 127.4 (C-2‴, C-6‴), 119.3 (C-4‴), 104.9 (C-4ʹʹ), 80.7 (C-1ʹ), 72.5 (C-5ʹ), 72.0 (C-3ʹ), 70.7 (C-2ʹ), 68.0 (C-4ʹ), 61.7 (C-6ʹ), 20.6‒20.1 (4 × *CH*_*3*_CO); ESI–MS (‒MS): *m/z* (%) 717.7 (M‒2H, 100); calc. for C_24_H_26_IN_5_O_11_S = 719.04 Da.

### 3-(2-Methyl-5-chlorophenyl)-4-formylsydnone *N*-(2,3,4,6-tetra-*O*-acetyl-β-d-glucopyranosyl)thiosemicarbazon (**4n**)

Dark yellow crystals, mp 122‒123 °C (from 96 % ethanol), R_f_ = 0.53; $[\alpha ]_{\text{D}}^{25}$ +43.2 (*c* = 0.22, CHCl_3_); FTIR (KBr): ν/cm^‒1^ 1754 (ν_C=O_ ester and sydnone), 3341, 3249 (ν_NH_), 1600 (ν_CH=N_), 1526, 1450 (ν_C=C_), 1080 (ν_C=S_), 1040, 1227 (ν_COC_ ester); ^1^H NMR (500 MHz, DMSO-*d*_6_): δ 12.20 (s, 1H, Hz, NH-2), 8.03 (d, 1H, *J* = 9.0 Hz, NH-4), 7.56 (s, 1H, CH = N), 7.70‒7.47 (m, 3H, H-3‴, H-4‴, H-6‴), 7.70‒7.47 (m, 2H, H-5‴, H-6‴), 5.97‒5.90 (m, 1H, H-1ʹ), 5.29 (t, 1H, *J* = 9.75 Hz, H-3ʹ), 5.12 (t, 1H, *J* = 9.75 Hz, H-4ʹ), 5.08‒5.02 (m, 1H, H-2ʹ), 4.30 (dd, 1H, *J* = 12.5, 4.5 Hz, H-5ʹ), 4.10-4.07 (m, 1H, H-6ʹb), 3.87 (s, 3H, 2‴-CH_3_), 3.84‒3.80 (m, 1H, H-6ʹa), 2.21–1.96 (s, 12H, 4 × *CH*_*3*_CO); ^13^C NMR (125 MHz, DMSO-*d*_6_): δ 179.6 (C = S), 170.9‒169.6 (4 × CH_3_*CO*), 166.4 (C-5ʹʹ), 139.8 (C-1‴), 131.9 (C-2‴), 132.4 (C-3‴), 126.4 (C-4‴), 132.9 (C-5‴), 129.9 (CH = N), 127.3 (C-6‴), 104.3 (C-4ʹʹ), 82.1 (C-1ʹ), 82.0 (C-2ʹ), 74.0 (C-5ʹ), 70.0 (C-3ʹ), 68.5 (C-4ʹ), 62.0 (C-6ʹ), 20.8‒20.4 (4 × *CH*_*3*_CO), 16.6 (2ʹʹ-CH_3_); ESI–MS (+MS): *m/z* (%) 642.02/644.03 (M + H/M + H+2, 65/25), 619.15 (14), 605.51 (6), 550.78 (10), 5232.91 (15), 474.38 (10), 462.39 (20), 448.45 (10), 430.52 (14), 414.45 (10), 374.37 (6), 335.48 (12), 296.77 (10), 267.57 (40), 240.37 (10), 139.54 (35), 117.58 (100), 102.52 (87), 81.39 (17), 54.25 (47); calc. for C_25_H_285_^35^ClN_5_O_11_S/C_25_H_28_^37^ClN_5_O_11_S = 641.12/643.11 Da.

### 3-Cyclohexyl-4-formylsydnone *N*-(2′,3′,4′, 6′-tetra-*O*-acetyl-β-d-glucopyranosyl)thiosemicarbazon (**4o**)

Dark yellow crystals, mp 126‒128 °C (from 96 % ethanol), R_f_ = 0.61; $[\alpha ]_{\text{D}}^{25}$ +44.0 (*c* = 0.21, CHCl_3_); FTIR (KBr): ν/cm^‒1^ 1756 (ν_C=O_ ester and sydnone), 3271, 2950 (ν_NH_), 1596 (ν_CH=N_), 1530–1378 (ν_C=C_), 1043 (ν_C=S_), 1043, 1223 (ν_COC_ ester); ^1^H NMR (500 MHz, DMSO-*d*_6_): δ 12.07 (s, 1H Hz, NH-2), 8.21 (d, 1H, *J* = 9.5 Hz, NH-4), 7.86 (s, 1H, CH=N), 5.97 (t, 1H, *J* = 9.5 Hz, H-1ʹ), 5.44 (t, 1H, *J* = 9.75 Hz, H-3ʹ), 5.29 (t, 1H, *J* = 10.5 Hz, H-1‴), 5.10 (t, 1H, *J* = 9.5 Hz, H-4ʹ), 4.93 (t, 1H, *J* = 9.75 HzH-2ʹ), 4.19 (dd, 1H, *J* = 2.0; 12.5 Hz, H-5ʹ), 4.11 (dd, 1H, *J* = 4.5, 12.5 Hz, H-6ʹa), 3.97 (d, 1H, *J* = 12.0 Hz, H-6ʹb), 2.20‒2.18 (m, 2H, 2 × H-3‴), 1.81‒1.74 (m, 2H, 2 × H-4‴), 1.71‒1.63 (m, 2H, 2 × H-5‴), 1.54‒1.52 (m, 2H, 2 × H-6‴), 1.29‒1.23 (m, 2H, 2 × H-2‴), 2.00‒1.95 (s, 12H, 4 × CH_3_CO); ^13^C NMR (125 MHz, DMSO-*d*_6_): δ 177.8 (C=S), 169.9‒169.3 (4 × CH_3_*CO*), 166.6 (C-5ʹʹ), 130.8 (CH=N), 101.5 (C-4ʹʹ), 81.2 (C-1ʹ), 72.5 (C-5ʹ), 72.3 (C-3ʹ), 70.8 (C-2ʹ), 67.8 (C-4ʹ), 63.6 (C-1‴), 61.7 (C-6ʹ), 30.6 (C-2‴), 30.0 (C-6‴), 24.5 (C-4‴), 24.1 (C-3‴), 24.0 (C-5‴), 20.4‒20.3 (4 × *CH*_*3*_CO); ESI–MS (‒MS): *m*/*z* (%) 598.3 (M‒H, 15), 559.1 (5), 459.2 (100), 431.4 (12); calc. for C_24_H_33_N_5_O_11_S = 599.19 Da.

### Antimicrobial screening

#### Antibacterial activity

The synthesized compounds **4a**–**o** were screened in vitro for their antibacterial activities against bacteria namely *Staphylococcus epidermidis* (ATCC 12228) and *Bacillus subtilis* (ATCC 6633) as Gram positive bacteria, *Escherichia coli* (ATCC 25922) and *Salmonella enterica* (ATCC 15442) as Gram negative bacteria, were tested by using agar well diffusion (cup-plate) method [32]. The sterilized nutrient agar medium was distributed 100 mL each and allowed to cool to room temperature. The 24 h old Mueller–Hinton broth cultures of test bacteria were swabbed on sterile Mueller–Hinton agar plates in sterilized Petri dishes using sterile cotton swab followed by punching wells of 6 mm with the help of sterile cork borer. The standard drug (ciprofloxacin, 1 mg/mL of sterile distilled water), compounds **4a**–**o** (500 μg/mL in 10 % DMSO, prepared by dissolving 2.5 mg of substance in 5 mL of 10 % DMSO solution in water), and control sample (a 10 % solution of DMSO in water) were added to the respectively labelled 6 mm diameter wells. The plates were allowed to stand for 30 min and then incubated at 37 °C for 72 h in upright position. When growth inhibition zones were developed surrounding each cup, their diameter in mm was measured and compared with that of ciprofloxacin (Table 3).

The antibacterial activities against above bacteria of all the synthesized derivatives also were evaluated in vitro by serial tube dilution method [33]. The compounds and standard drug ciprofloxacin were dissolved in DMSO to give a concentration of 5 μg/mL (stock solution). A set of test tubes of capacity 5 mL was washed, cleaned and dried completely. Double strength nutrient broth was used as a growth/culture media for all bacteria. The culture media was made by dissolving 15 g of nutrient broth No. 2 in 1 L of distilled water. Approximately 1 mL of this culture media was prepared and transferred to each test tube by micropipette and capped with non-adsorbent cotton plugs. A set of test tubes containing 1 mL culture media was sterilized in an autoclave at 15 psi pressure at 121 °C for 20 min. Sub-culturing of bacteria was done by transferring a loopful of particular bacterial strain from standard bacterial agar slant to 10 mL sterilized nutrient broth aseptically in a laminar air flow cabinet. It was then incubated for a period of 24 h at 37 °C in a incubator. After 24 h incubation the bacterial stain suspension was prepared by aseptically inoculating 0.2 mL of revived bacterial colony into 100 mL of 0.9 % m/v saline. The study involved a series of five assay tubes for each compound against each strain. A stock solution of each test compound at concentration 5 μg/mL was serially diluted in series of 5 assay test tubes (containing 1 mL nutrient broth) to give concentration of 2.5, 1.25, 0.625, 0.313 and 0.156 μg/mL. Then, 0.1 mL of normal saline suspension of revived bacteria was added to each test tube. The inoculated tubes were incubated at 37 °C for 24 h. The MIC (minimum inhibitory concentration) values were determined by subsequently checking for the absence of visual turbidity (Table 4).

Experiments were repeated three times, and the results were expressed as average values.

#### Antifungal activity

The synthesized compounds **4a**–**o** were screened for their antifungal activity against three fungal strains [34], namely *Aspergillus niger* 439, *Candida albicans* ATCC 7754, *Fusarium oxysporum* M42, at the concentration levels of 500 μg/mL (Table 4) by agar well diffusion (cup-plate) method, using nystatin as the standard and control sample is a 10 % solution of DMSO in water. The sterilized potato dextrose agar medium incubated at 30 °C for 48 h, then the subculture of fungus were added, and shaken thoroughly to ensure uniform distribution. After that, this was poured into previously sterilized and labelled Petri dishes and allowed to solidify. Two cups were filled with 0.1 mL of two test dilutions and the other two cups with respective concentrations of standard dilutions. The plates were left as it is for 2–3 h for diffusion and then they were kept for 24 h at 37 °C for incubation. Then the diameter of the zones of growth inhibition was measured and compared with that of standard (nystatin).

Similarly, the antifungal activities against above fungi of all thiosemicarbazone derivatives also were evaluated in vitro by serial tube dilution method [33, 34]. Experiments were repeated three times, and the results were expressed as average values.

## Abbreviations

OAc: acetyl
DMF: *N*,*N*-dimethylformamide
DMSO: dimethyl sulfoxide
diMe: dimethyl
FTIR: Fourier-transformed infrared spectroscopy
MS: mass spectrometry
NMR: nuclear magnetic resonance spectroscopy
ESI: electron-spray ionization
